# The evolution of trypanosomatid taxonomy

**DOI:** 10.1186/s13071-017-2204-7

**Published:** 2017-06-08

**Authors:** Alexa Kaufer, John Ellis, Damien Stark, Joel Barratt

**Affiliations:** 1 School of Life Sciences, University of Technology Sydney, Ultimo, NSW 2007 Australia; 2 Department of Microbiology, St Vincent’s Hospital Sydney, Darlinghurst, NSW 2010 Australia

**Keywords:** *Leishmania*, *Leptomonas*, *Zelonia*, Trypanosomatid, Taxonomy, Phylogenetics, Systematics

## Abstract

Trypanosomatids are protozoan parasites of the class Kinetoplastida predominately restricted to invertebrate hosts (i.e. possess a monoxenous life-cycle). However, several genera are pathogenic to humans, animals and plants, and have an invertebrate vector that facilitates their transmission (i.e. possess a dixenous life-cycle). *Phytomonas* is one dixenous genus that includes several plant pathogens transmitted by phytophagous insects. *Trypanosoma* and *Leishmania* are dixenous genera that infect vertebrates, including humans, and are transmitted by hematophagous invertebrates. Traditionally, monoxenous trypanosomatids such as *Leptomonas* were distinguished from morphologically similar dixenous species based on their restriction to an invertebrate host. Nonetheless, this criterion is somewhat flawed as exemplified by *Leptomonas seymouri* which reportedly infects vertebrates opportunistically. Similarly, *Novymonas* and *Zelonia* are presumably monoxenous genera yet sit comfortably in the dixenous clade occupied by *Leishmania*. The isolation of *Leishmania macropodum* from a biting midge (*Forcipomyia* spp.) rather than a phlebotomine sand fly calls into question the exclusivity of the *Leishmania*-sand fly relationship, and its suitability for defining the *Leishmania* genus. It is now accepted that classic genus-defining characteristics based on parasite morphology and host range are insufficient to form the sole basis of trypanosomatid taxonomy as this has led to several instances of paraphyly. While improvements have been made, resolution of evolutionary relationships within the Trypanosomatidae is confounded by our incomplete knowledge of its true diversity. The known trypanosomatids probably represent a fraction of those that exist and isolation of new species will help resolve relationships in this group with greater accuracy. This review incites a dialogue on how our understanding of the relationships between certain trypanosomatids has shifted, and discusses new knowledge that informs the present taxonomy of these important parasites.

## Background

The Trypanosomatidae are a diverse family of protozoan parasites that are predominately monoxenous. Nonetheless, some trypanosomatids occupy a dixenous (syn. digenetic) niche [1]. *Phytomonas* is one example of a dixenous trypanosomatid genus, transmitted by phytophagous insects and parasitising a variety of plants. However, the trypanosomatids were brought to prominence by two genera, *Trypanosoma* and *Leishmania*, attributable to their role as human pathogens. *Trypanosoma* and *Leishmania* are obligatorily dixenous, possess zoonotic or anthroponotic life-cycles, and are transmitted by hematophagous insects. These parasites cause devastating human diseases including Human African Trypanosomiasis (aetiology: *Trypanosoma brucei*), Chagas disease (aetiology: *Trypanosoma cruzi*), and the leishmaniases, which are attributable to roughly 20 species of *Leishmania* [2–8] (Table 1). These diseases are associated with a spectrum of symptoms differing in severity and mechanisms of pathogenesis [8]. These dixenous species also differ in their preferred site of development within their vertebrate hosts which influences disease phenotype and severity [9–11]. With the exception of the *gambiense* form of Human African Trypanosomiasis (HAT) and infections caused by *Leishmania tropica* and *Leishmania donovani*, for which humans are considered the main reservoir, trypanosomatid-associated diseases are predominately zoonotic, with animal reservoirs playing a key role in maintaining endemicity [8, 12].

**Table 1:** Currently recognised genera of the family Trypanosomatidae

Genus	Invertebrate host	Description
Monoxenous		
* Angomononas*	*Zelus leucogrammus*, *Ornidia obesa*, *Chrysomya putoria*, *Chrysomya megacephala*, *Lucilia cuprina*	Genus within the subfamily *Strigomonadiae*. Members of this genus harbour an obligate intra-cytoplasmic beta-proteobacterial symbiont [57]. *Angomonoas deanei* (formerly *Crithidia deanei*), the type-species, has a small, rounded choanomastigote with truncated anterior ends.
* Blastocrithidia*	*Gerris remiges*, *Euschistus servus*	The genus is characterised by the epimastigote form with its pointed ends and the anterior location of the kinetoplast [56]. *Blastocrithidia triatome* infects reduviid bugs (the vector of *T. cruzi*), reducing their life span and reproduction rate [134]. Other species include *Blastocrithidia gerridis*, commonly found in *Gerris remigis* “water striders” and *Blastocrithidia euschisti* in the *Euschistus servus* “milkweed” bug [56].
* Blechomonas*	*Pulex irritans*, *Chaetopsylla* spp. *Ctenophthalmus* spp., *Monopsyllus sciurorum*, *Paraceras melis*, *Ceratophyllus* spp., *Nosopsyllus fasciatus*, *Ctenocephalides* spp., *Nycteridopsylla* spp., *Archeopsylla erinacei*	A relatively new genus reserved for species that are strictly found in fleas (Siphonaptera) [135]. Morphologically, *Blechomonas* is a diverse genus, comprising of extremely pleomorphic cells including promastigotes, choanomastigotes and amastigotes.
* Crithidia*	*Bombus hortorum*, *Bombus muscorum*, *Bombus terrestris*, *Culex* spp*.*	This genus includes common parasites of the insect alimentary canal. They possess small, wide cell bodies with a truncated anterior end and broad posterior end [56]. *Crithidia bombi* is the most extensively studied species given its potential role in the reduction of bumblebee reproductive fitness. It infects several bee species: *Bombus terrestris*, *Bombus muscorum* and *Bombus hortorum* [136]. *Crithidia mellificae* is a honeybee pathogen associated with colony losses and *Crithidia fasciculate* infects mosquitoes.
* Herpetomonas*	*Musca domestica*	A genus that includes a variety of morphological types including promastigote and opisthomastigote forms. *Herpetomonas* is predominately found in dipterans, with *Herpetomonas muscae domesticae*, the type-species, found in the common housefly [95]. Members of the *Herpetomonas* genus have also been detected in hemipterans, plants and a rat [95].
* Kentomonas*	*Sarcophaga* (*sensu lato*) sp.	Another novel endosymbiont-harbouring trypanosomatid genus within the subfamily Strigomonadiae [133].
* Leptomonas*	*Proba sallei*, *Collaria oleosa*, *Neotropicomiris nordicus*, *Hyalymenus* sp*.*, *Jadera aeola aeola*, *Camptischium clavipes*, *Dysdercus* spp*.*, *Calocorisca altiplana*, *Stenodema andina*, *Prepops* cf. *accinctus*	Trypanosomatids with a life-cycle containing both promastigote- and amastigote stages [76], parasitic only in invertebrates and generally considered of no medical importance [77–79]. However, several reports of *Leptomonas seymouri* infection in vertebrates have emerged [53].
* Lotmaria*	*Apis mellifera*	A novel clade in the subfamily Leishmaniinae that infects the honey bee, *Apis mellifera* [137]. *Crithidia mellificae* was once considered the predominant trypanosomatid of the honey bee. Phylogenetics facilitated reassignment of some *Crithidia* parasites to the newly described *Lotmaria passim*. While *C. mellificae* is still extant in bee populations *Lotmaria passim* is the most prevalent trypanosomatid in *A. mellifera* [137].
* Novymonas*	*Niesthrea vincentii*	A newly established genus accommodating a novel endosymbiont-bearing trypanosomatid that exist aspredominantly as promastigotes and choanomastigotes [87].
* Paratrypanosoma*	*Culex pipiens*	This genus represents the missing link between the free-living bodonid family and the parasitic trypanosomatids [83]. *Paratrypanosoma confusum*, predominately exists as an elongated promastigote in the intestine of female mosquitoes (Diptera: Nematocera: Culicidae).
* Sergeia*	*Culicoides festivipennis*, *Culicoides truncorum*	This genus is represented by the novel endosymbiont-free *Sergeia podlipaevi* [138]. In the midgut of their hosts, they exist as promastigotes with the nucleus located in the centre or proximal to the posterior portion of the cell.
* Strigonomonas*	*Aedes vexans*, *Oncopeltus* sp*.*, *Lutzomya almerioi*	This genus is comprised of endosymbiont-bearing trypanosomatids also of the *Strigomonadiae* subfamily characterised by flagellates of diverse shapes and length. *Strigomonas oncopelti* (syns *Herpetomonas oncopelti*, *Leptomonas* (*Strigomonas*) *oncopelti*, *Crithidia oncopelti*), the type-species, harbour an obligate intra-cytoplasmic betaproteobacterial symbiont [57].
* Wallaceina*	*Nabis brevis*, *Nabis flavomarginatus*, *Calocoris sexguttatus*	This genus was established to incorporate trypanosomatids that produce endomastigotes. The taxonomy of this genus is somewhat confusing as upon its establishment to accommodate the newly discovered *Wallaceina inconstans*, *Crithidia brevicula* was moved into this genus. Few isolates have been discovered that possess phylogenetic affinity to *Wallaceina* [139].
* Zelonia*	*Simulium* (*Morops*) *dycei*, *Ricolla simillima*	A genus created to accommodate the trypanosomatid previously named *Leptomonas costaricensis* [28]. Along with the newly described *Zelonia australiensis*, this genus includes parasites that are immediately basal to the dixenous clade occupied by *Leishmania*, *Endotrypanum* and *Porcisia*.
Dixenous		
* Endotrypanum*	*Phlebotomus* spp.	A genus comprised of species that infect the erythrocytes of their mammalian hosts, which include the Neotropical tree sloths (genera *Choloepus* and *Bradypus*). *Endotrypanum* is comprised of the species *E. schaudinni* and *E. monterogeil* as well as three species previously placed in the genus *Leishmania* including *E. herreri*, *E. colombiensis* and *E. equatorensis* [28].
* Leishmania*	*Phlebotomus* spp., *Lutzomyia* spp., *Forcipomyia* (*Lasiohelea*) spp.	*Leishmania* species were once differentiated by their respective proliferative stages [18]. Female phlebotomine sand flies are the natural vectors for *Leishmania* transmission, and roughly 70 known animal species serve as reservoirs for human pathogenic *Leishmania* species, including rodents [20], dogs [21] and other mammals [22]. Approximately 20 species of *Leishmania* act as the aetiological agents of human leishmaniasis.
* Phytomonas*	*Phthia picta*, *Nezara viridula*, *Oncopeltus fasciatus*	*Phytomonas* is a dixenous genus that includes several plant pathogens transmitted by phytophagous insects [140]. They predominately exist as promastigotes and less frequently as choanomastigotes [47]. *Phytomonas* spp. have been isolated from a wide-range of plant tissues including the fruit, flower, seeds and the phloem [51].
* Porcisia*	Vector unknown	A new genus recently established to accommodate the Neotropical porcupine-infecting parasites previously known as *Leishmania hertigi* and *Leishmania deanei* [28].
* Trypanosoma*	*Triatoma infestans*, *Rhodnius prolixus*, *Glossina* spp*.*	*Trypanosoma* species infect reptiles, fish, birds and mammals, including humans, and are transmitted by hematophagous insects and aquatic leeches [141, 142]. Certain members of this genus cause devastating human diseases including Human African Trypanosomiasis (aetiology: *Trypanosoma brucei*) and Chagas disease (aetiology: *T. cruzi*) [13].

**Fig. 1:**
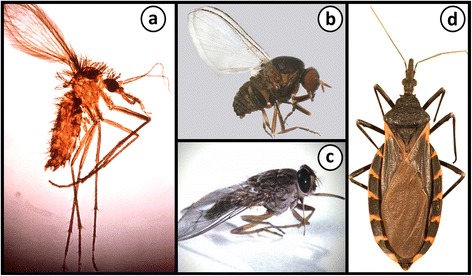
Vectors and invertebrate hosts of some trypanosomatids. **a **A female *Phlebotomus *sp. sand fly which is a vector of *Leishmania* spp. Citation: Hailu et al. Visceral leishmaniasis: New health tools are needed. PLoS Med. 2005;2(7):590–594 [151]. **b** A female *Simulium* (*Morops*) *dycei*, which is the host of *Zelonia australiensis*. Citation: Barratt et al. Isolation of novel trypanosomatid, *Zelonia australiensis* sp. nov. (Kinetoplastida: Trypanosomatidae) provides support for a Gondwanan origin of dixenous parasitism in the Leishmaniinae. PLOS Negl Trop Dis. 2017;11(1):e0005215 [68]. **c** The tsetse fly is the vector of *Trypanosoma brucei*; the aetiological agent of Human African Trypanosomiasis (http://researchnews.plos.org/2016/08/08/under-my-skin/) [152]. **d** A Triatomine “kissing” bug, which is the natural vector of *Trypanosoma cruzi*; the aetiological agent of Chagas disease. Citation: Curtis-Robles et al. Combining public health education and disease ecology research: using citizen science to assess Chagas disease entomological risk in Texas. PLoS Neglect Trop Dis. 2015;9(12):12 [153]. **a**, **b**, **d** Copyright: Creative Commons Attribution 4.0 International License (https://creativecommons.org/licenses/by/4.0/)

*Trypanosoma* spp. are ubiquitous, infecting almost all vertebrate classes [13], with vectors ranging from leeches, to biting flies and bugs [8, 14]. The tsetse fly (genus: *Glossina*) and bugs of the Triatominae subfamily (i.e. “kissing” bugs) are the natural vectors of *Trypanosoma brucei* and *Trypanosoma cruzi*, respectively [8] (Fig. 1). Human African Trypanosomiasis is endemic in 36 sub-Saharan countries with studies estimating that 61 million people are at risk of contracting the disease through the bite of an infected tsetse fly [15]. Domestic and wild animals serve as reservoirs for *T. brucei* infection, with the reported human prevalence varying between communities [2]. Between 2004 and 2014, the number of new cases reported has significantly dropped from 17,616 to only 3,796 with the total number of estimated annual cases plummeting from 50,000–70,000 to less than 15,000 [15]. Chagas disease is endemic in Central and South America, with an estimated 8 million individuals infected [16]. Humans are an incidental host of *Trypanosoma cruzi*, which is a zoonotic parasite predominantly infecting native wildlife, domestic dogs and cats, which are reservoirs of Chagas disease [17].

Female phlebotomine sand flies (Diptera: Psychodidae: Phlebotominae) are natural vectors for *Leishmania* transmission [18, 19], and roughly 70 animal species serve as reservoirs for human pathogenic *Leishmania* species, including rodents [20], dogs [21] and other mammals [22]. Australia and Antarctica had long been considered the only continents free of endemic *Leishmania* though the discovery of an Australian macropod-infecting species and its midge vector, *Forcipomyia* (*Lasiohelea*) spp. [20, 22, 23], overturned this and called into question the exclusivity of the sand fly-*Leishmania* interaction.

Compared to their dixenous cousins, the life-cycle and habits of monoxenous trypanosomatids are obscure. By 2001, monoxenous trypanosomatids had been identified from roughly 350 insect species only, while more than 900 vertebrate hosts had been identified for the dixenous genera which are far fewer in number [24]. Due to their limited impact on human and animal health, the monoxenous trypanosomatids have received little attention from parasitologists historically. Despite this, interest in the monoxenous species has revived in recent years [25–28]. From a taxonomic perspective, trypanosomatids are now amongst the most extensively studied protozoans, as reflected by the recent surge in publications on this topic that form the basis of the trypanosomatid taxonomic system [26, 27, 29].

**Fig. 2:**
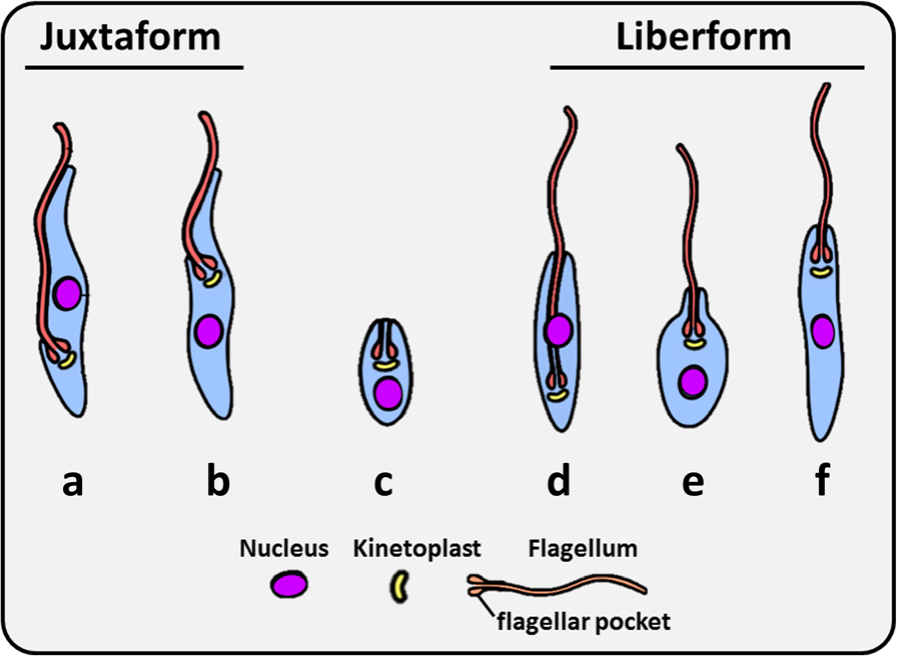
The six major morphotype classes of trypanosomatids. **a** Trypomastigote. **b** Epimastigote. **c** Amastigote. **d** Opisthomastigote. **e** Choanomastigote. **f** Promastigote. Forms **a** and **b** represent the juxtaform superclass and possess a flagellum that is laterally attached to the cell body. Forms **d**, **e** and **f** represent the liberform superclass and do not possess a laterally attached flagellum. Amastigotes (**c**) exist for both liberform and juxtaform trypanosomatids

Trypanosomatid systematics was traditionally based on host preferences and specialised life-cycle stages, characterised by the presence or absence of several defined morphotypes (Fig. 2) [30–32]. Under the classical system of trypanosomatid taxonomy, the various *Leishmania* species were differentiated by their respective specialised proliferative stages [18]. Advances in molecular biology revolutionised the field by providing genetic evidence for true ancestral relationships [27, 33]. As a consequence, major flaws were identified in the classical system which underwent a series of major revisions [26–29]. However, the field would continue to benefit from studies aiming to isolate new species. Of the estimated one million known insect species described, only 2,500 of these have been studied closely for the presence of trypanosomatids [24, 26, 29, 34]. The isolation and molecular characterisation of new species will help to decipher relationships between trypanosomatids with greater resolution [24].

## Development of *Leishmania* and *Trypanosoma* in vertebrates

**Fig. 3:**
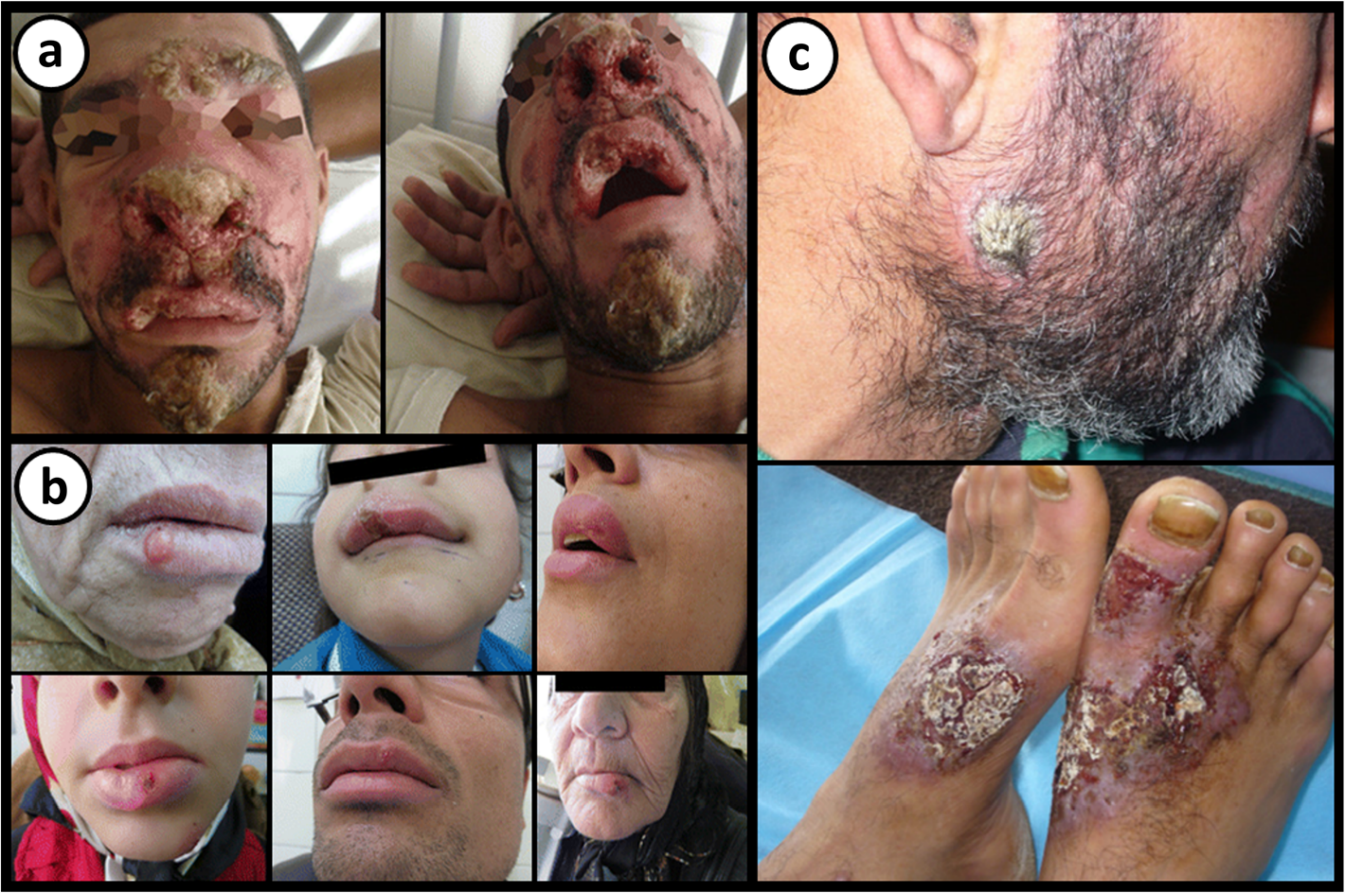
Some clinical manifestations of leishmaniasis. **a** A patient with mucocutaneous leishmaniasis (MCL) presenting with facial ulcerative lesions and nasal obstruction. Cropped from original. Citation: Gois et al. Immune response to *Leishmania* antigens in an AIDS patient with mucocutaneous leishmaniasis as a manifestation of immune reconstitution inflammatory syndrome (IRIS): a case report. BMC Infect Dis. 2015;15(1):38 [154]. **b** Presentation of MCL with patients suffering from erythematous papules and ulcerations on the lip region. Cropped from original. Citation: Mohammadpour et al. Lip leishmaniasis: a case series with molecular identification and literature review. BMC Infect Dis. 2017;17(1) [155]. **c** A patient with cutaneous leishmaniasis presenting with crusted nodules over the left cheek (upper panel) and erythematous ulcerated plaques with crusts over the feet (lower panel). Cropped from original. Citation: Al-Dwibe et al. Contact dermatitis-like cutaneous leishmaniasis in a Libyan HIV patient. Parasit Vectors. 2014;7:3 [156]. **a**-**c** [157]

**Fig. 4:**
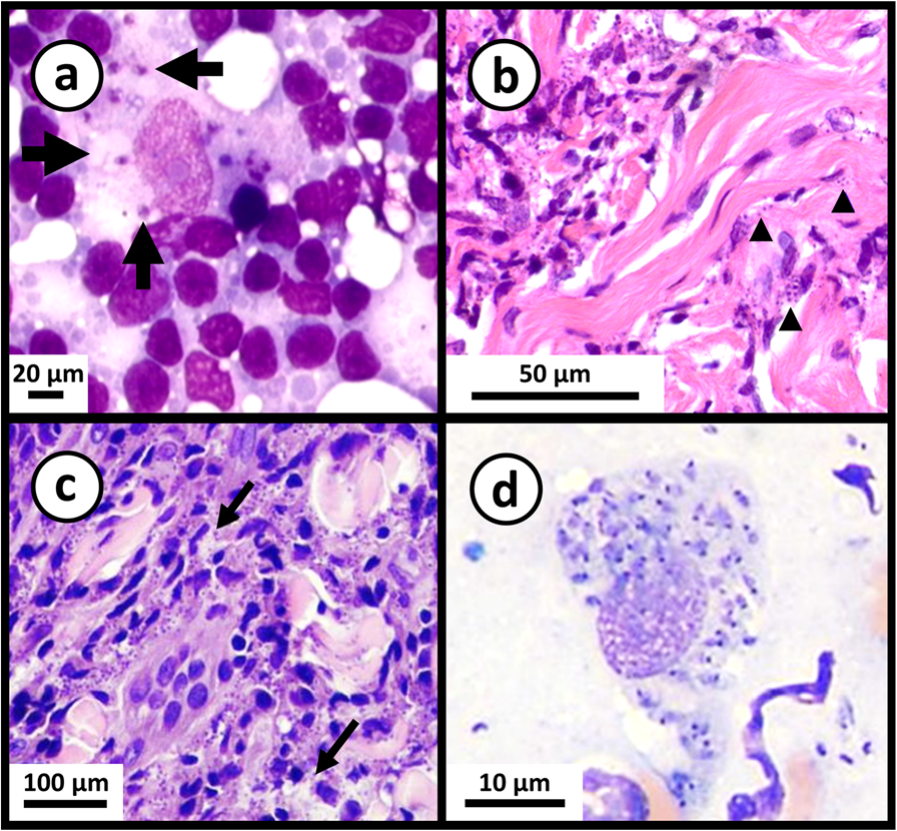
Photomicrographs of stained smears showing *Leishmania* infections. a May-Grunwald-Giemsa stained preparation from a case of feline leishmaniasis showing macrophages infected with *L. infantum* amastigotes. Cropped from original. Citation: Pennisi et al. [157]. LeishVet update and recommendations on feline leishmaniosis. Parasit Vectors. 2015;8(1):302. **b** Haematoxylin and eosin stained histological preparation from a canine deep dermis mucocutaneous lesion showing *Leishmania* amastigotes and *Leishmania-*infected fibroblasts (arrowheads). Cropped from original. Citation: Baneth et al. [158]. Mucocutaneous *Leishmania tropica *infection in a dog from a human cutaneous leishmaniasis focus. Parasit Vectors. 2014;7:5. **c** Haematoxylin and eosin stained preparation from a canine with cutaneous leishmaniasis showing intracellular *Leishmania* amastigotes in macrophages (arrows). Cropped from original. Citation: Ordeix et al. [159]. Histological and parasitological distinctive findings in clinically-lesioned and normal-looking skin of dogs with different clinical stages of leishmaniosis. Parasit Vectors. 2017;10:8. **d** May-Grunwald-Giemsa stained preparation from an aspirate of a mucocutaneous lesion predominately showing intracellular *Leishmania* amastigotes and few extracellular amastigotes. Cropped from original. Citation: Baneth et al. [158]. **a**-**d** Copyright: Creative Commons Attribution 4.0 International License (http://creativecommons.org/licenses/by/4.0/)

During a blood meal the infected sand-fly injects metacyclic promastigotes into the vertebrate host [34]. In the vertebrate host, these promastigotes invade macrophages and then differentiate into non-motile amastigotes, which multiply by binary fission [34]. *Leishmania* replicates within macrophages at various sites depending on the species [10], giving rise to three distinct clinical forms of leishmaniasis; cutaneous leishmaniasis (CL), mucocutaneous leishmaniasis (MCL) and visceral leishmaniasis (VL or Kala Azar) [4]. These clinical forms result from parasite development within the reticuloendothelial system of either the skin (CL), nasopharynx (MCL) or viscera (VL) [35] (Figs. 3, 4).

CL is the most common clinical form of the disease, presenting as a myriad of manifestations affecting the skin [8]. It is characterised by the presence of one or more skin lesions with varying morphologies that can result in highly disfiguring scars [8]. Mucocutaneous leishmaniasis is a severely disfiguring form of leishmaniasis with 90% of MCL patients having suffered from a case of CL that had healed 1–5 years prior to the onset of MCL [36]. MCL involves the ulceration of the nasal region, accompanied by symptoms of fever and hepatomegaly [36]. Later progression involves ulceration to the oronasopharyngeal mucosa, resulting in erythema and edema of the affected regions. VL is the most severe manifestation of the disease [8]. VL is a systemic disease, characterised by the incidence of irregular bouts of fever, significant weight loss, splenomegaly and anaemia [8]. If untreated, fatality is almost certain as a result of haemorrhaging or co-infections with bacteria or viruses [4].

Human infective trypanosomes differ significantly in their route of infection, proliferative stages, and preferred sites of infection. *Trypanosoma cruzi* is transmitted in the faeces of their triatomine vector which defecates on the victim’s skin during a blood meal. The action of biting causes the victim to unknowingly scratch the bite area rubbing metacyclic trypomastigotes of *T. cruzi* from the faeces into the bite wound or into micro-abrasions caused by scratching [37]. These motile metacyclics invade host cells where they differentiate into amastigotes [4]. These intracellular amastigotes multiply by binary fission, causing tissue damage and host cell apoptosis [38]. *Trypanosoma cruzi* favours cells of the cardiovascular system, though also affects cells of the nervous and muscular reticuloendothelial systems [3, 39]. Additionally, the oral mode of *T. cruzi* transmission is emerging as a major route of infection for humans and animals in some endemic regions [40]. Oral transmission occurs *via* the ingestion of food contaminated with infected triatomine bugs or their faeces. Between 1968 and 2000, 50% of acute cases of Chagas disease recorded from the Brazilian Amazon were attributed to micro-epidemics of the disease caused by oral transmission [40]. Experimental evidence indicates that the infectivity of metacyclic trypomastigotes increases upon contact with gastric juices, a process that is thought to be modulated by certain trypomastigote surface glycoproteins [40, 41]. Alternatively, recent rodent studies suggest that the membranes of the oral mucosa are the most important site of *T. cruzi* entry into the host *via* the oral route [42].

Chagas disease has two phases, an acute and chronic phase [5]. In most cases of acute infection, patients are asymptomatic or may experience a wide-range of general symptoms including fever, headaches, heart inflammation, difficulty in breathing, diarrhoea and enlarged lymph glands [16]. Twenty to forty percent of those infected will suffer from severe, irreversible complications during the chronic phase including fatal cardiomyopathy, gastrointestinal and neurological problems [16, 17]. It is important to note that recently, *T. cruzi* has been divided into six discrete taxonomic units (DTUs) which represent distinct lineages [43, 44]. Recent studies suggest that these DTUs differ in their geographical distribution, transmission and clinical manifestation [44], implying that *Trypanosoma cruzi* represents a species complex.

Transmission of *T. brucei* occurs through the bite of the tseste fly, where metacyclic trypomastigotes are injected into the bite wound [4]. Following infection, *T. brucei* metacyclics transform into blood stream trypomastigotes where they undergo multiplication by binary fission, travelling throughout the blood stream and lymphatic system [45]. Unlike *T. cruzi*, *T.* remains extracellular throughout its entire life-cycle.

Human African Trypanosomiasis has two distinct forms of infection, chronic and acute, which are caused by two distinct subspecies of *T. brucei* [46]. The chronic form (aetiology: *Trypanosoma brucei gambiense*)*,* is endemic in western and central Africa and is the most common form of Human African Trypanosomiasis (HAT), with humans considered the main reservoir for the disease [12]. The acute infection (aetiology: *Trypanosoma brucei rhodesiense*)*,* is endemic in eastern and southern Africa and is predominately a zoonotic disease that occasionally affects humans [12]. The clinical manifestations of both acute and chronic HAT are often similar but vary in incubation period and severity. During the early stages of infection the parasites reside in the lymphatic system and blood stream causing fever, general malaise, weakness, lymphadenopathies, endocrine disturbances, musculoskeletal pains, and hepatosplenomegaly [12]. In the acute *rhodesiense* form, the early stage is often fatal as one tenth of patients do not have access to treatment and die from myocardial involvement as a consequence [46]. In the *gambiense* form, early stage symptoms are often non-specific including lymphadenopathy and hepatosplenomegaly [46]. The second, later stage of infection occurs following an incubation period of weeks and months in *rhodesiense* and *gambiense* infection, respectively*.* In this stage of the infection, the blood–brain-barrier is compromised, allowing the movement of parasites into the brain where it causes severe neurological manifestation including chronic encephalopathy and finally, coma and death [46].

## Development of *Phytomonas* in plants

The genus *Phytomonas* includes several plant pathogens that are transmitted by phytophagous insects. The biology and life-cycle of *Phytomonas* spp. is a poorly understood area of science. *Phytomonas* has been isolated from 24 different plant families, where they primarily occur as promastigotes and less frequently as choanomastigotes (Fig. 2) [47], while it assumes the form of a slim promastigote in the insect vector [48]. *Phytomonas* species can infect more than 100 plant species including lactiferous plants, tomato fruits, the coffee tree, coconut and oil palms [49, 50]. *Phytomonas* spp. have been isolated from a wide-range of tissues including the fruit, flower, seeds and phloem of plants [51]. The genus *Phytomonas* is restricted to trypanosomatids infecting plants [52], though the ability of *Leptomonas seymouri* to multiply in plants following experimental infections suggests that this taxonomic criterion should be used with caution and highlights the requirement for molecular evidence when making taxonomic assignments [53].

## Development in invertebrates

### Monoxenous trypanosomatids

**Fig. 5:**
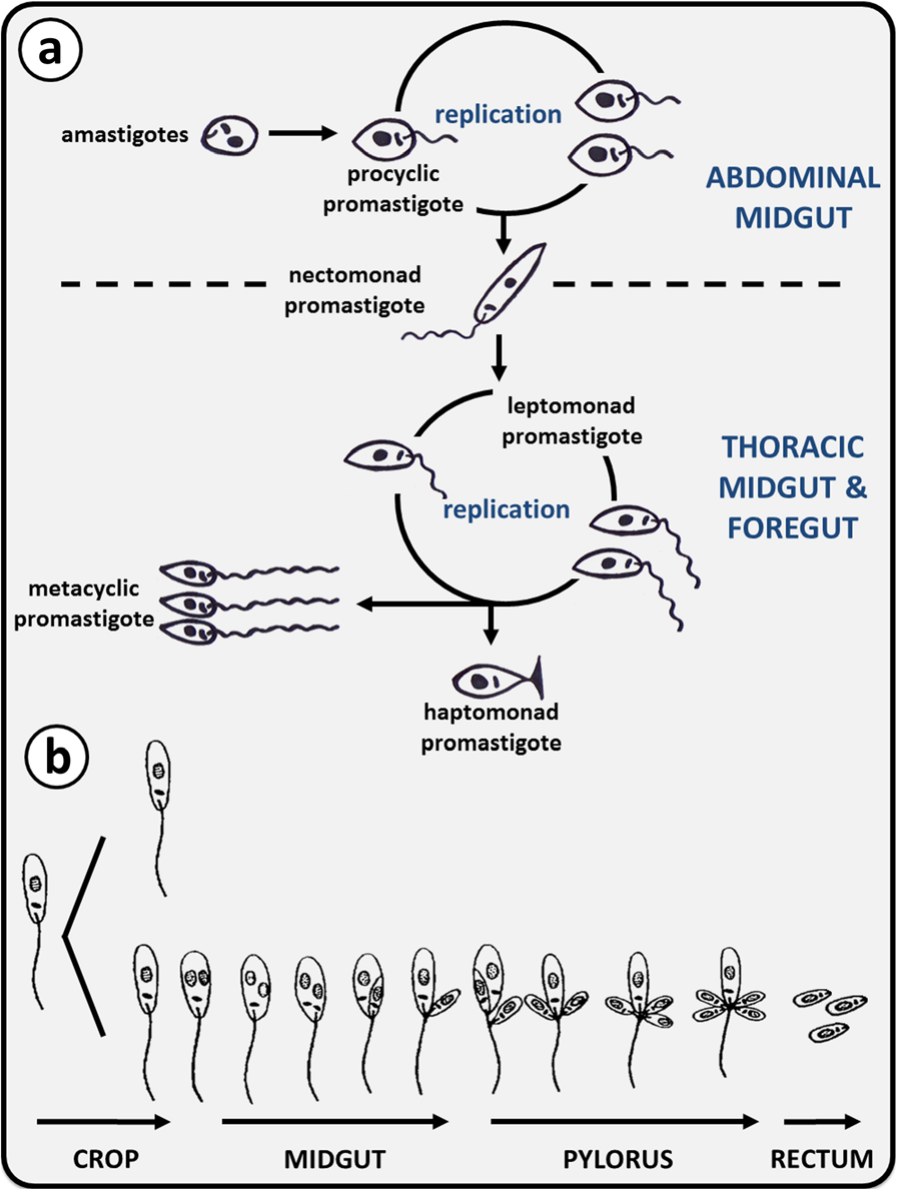
The growth cycle of trypanosomatids within invertebrates. **a** Replication of *Leishmania* (*Leishmania*) species in the sand fly vector occurs at two locations: procyclic promastigotes replicate in the abdominal midgut and leptomonad promastigotes in the thoracic midgut. The replicative procyclic promastigote forms differentiate into elongated nectomonad promastigotes that migrate anteriorly into the thoracic midgut, where further replication in the leptomonad form occurs. Some leptomonad promastigotes attach to the cuticle-lined surface of the midgut and differentiate into haptomonad promastigotes that may act as precursors for differentiation into metacyclic promastigotes, which is the stage infective to the mammalian host. **b** *Leptomonas* are ingested in the cyst form and differentiate into the promastigote form. In the crop, the leptomonad form undergoes fission and later in the midgut and pylorus by unequal fission or budding. Cysts are formed *via* budding in the rectum and are passed out in the faeces as the infective form

The monoxenous trypanosomatids infect a broad range of insects, including those of the orders Diptera, Hemiptera, Hymenoptera and Siphonaptera [54]. The life-cycle of monoxenous trypanosomatids has only been described for a few species, and current knowledge is based namely on the development of *Leptomonas ctenocephali* in the flea [55], and *Strigomonas oncopelti* from the spotted milk-weed bug [56]. *Strigomonas oncopelti* was originally named *Leptomonas oncopelti* based on the classical trypanosomatid taxonomic system, though was reassigned to the genus *Strigomonas* based on phylogenetic analysis [26, 57]. Invertebrate hosts of monoxenous trypanosomatids may become infected *via* multiple routes including ingestion of cyst-like amastigotes from the faeces of another infected hosts [56], food sharing, predating other infected insect species, or cannibalism [58]. In the posterior portion of the invertebrate (i.e. the insect hindgut), reproduction occurs in one of two ways: binary fission or budding. The former type of reproduction involves nuclear division of promastigotes resulting in daughter cells equal to that of the parent organism. Budding is the mode of reproduction employed by amastigotes, which multiply in the rectum until they are excreted in the faeces [55, 56, 59]. It should be noted that this description of the monoxenous life-cycle represents a generalisation based on knowledge of only a few monoxenous species, and may not apply to all (Fig. 5).

### *Trypanosoma*

*Trypanosoma* species employ one of two methods of development within their invertebrate host, termed Salivaria and Stercoraria [60]. Salivaria as observed in *T. brucei* and other African trypanosomes, is characterised by development within the frontal portion of the invertebrates’ digestive system and transmitted through the bite of an insect [61]. Stercoraria as observed in *Trypanosoma cruzi*, is characterised by development of parasites within the posterior region of the invertebrates’ hindgut and transmitted through the excretion of faeces [60]. *Trypanosoma evansi*, the aetiological agent of the livestock disease Surra, is an example of a trypanosome that is transmitted mechanically [62]. As a result of the loss of mincircles and maxicircles of the kinetoplast DNA [63], *T. evansi* cannot undergo replication in the tsetse fly and is mechanically transmitted between vertebrates in the mouthparts of blood-feeding flies including the tabanids and stomoxes *Trypanosoma equiperdum* is unusual amongst trypanosomatids as it is sexually transmitted between horses, causing an illness known as Dourine [62].

### *Leishmania*

**Fig. 6:**
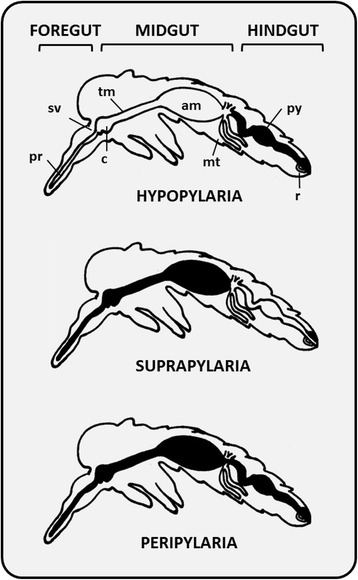
Diagrammatic representation of the three *Leishmania* sections proposed by Lainson & Shaw (1979). Figure shows the sections Hypopylaria, Suprapylaria and Peripylaria relative to the relevant structural features of the sand fly including the proboscis (pr), stomodeal valve (sv), cardia (c), thoracic midgut (tm), abdominal midgut (am), malpighian tubules (mt), pylorus (py) and rectum (r). The distribution of *Leishmania* development within the sand fly vector is shown in *black*

Through the detailed study of *Leishmania* development in sand flies it became increasingly clear that this process was not uniform across the genus. There are several morphological classes of promastigotes that were once used for taxonomic purposes including procyclic, nectomonad, leptomonad, haptomonad and metacyclic promastigotes, discussed later in this paper. A landmark suggestion by Lainson et al. [64] in 1979 established three ‘sections’ discernible by differences in their site of development within the sand fly. These sections greatly aided the classification of *Leishmania*, and are as follows: (i) Hypopylaria; development in the invertebrate hindgut (restricted to some species that infect only reptiles, i.e. some saurian species), (ii) Peripylaria; development in the hindgut and pylorus, and (iii) Suprapylaria; development anterior to the invertebrate pylorus [65] (Fig. 6). In 1987 species in the Suprapylaria section were assigned to the subgenus *Leishmania*, i.e. *Leishmania* (*Leishmania*), and those in the Peripylaria section were assigned to the subgenus *Viannia* [19]. This system proved to be an excellent taxonomic criterion, as the three sections were phylogenetically supported [19]. The Hypopylaric *Leishmania* species are also restricted to lizards; a convenient taxonomic criterion that supports this section, leading to establishment of the genus *Sauroleishmania* which was eventually demoted to subgeneric status [19, 66]. Debate over which section was the more primitive ensued to decide which section should occupy the root of phylogenetic trees with the Hypopylaric (subgenus *Sauroleishmania*) and Peripylaric (subgenus *Viannia*) species representing the prime contenders [66, 67]. Phylogenetic evidence indicates that Hypopylaria does not reflect the most primitive state as saurian species occupy a position closer to the crown of trees than *Viannia* [68]. In fact, the subgenus *Mundinia* is the most basal subgenus of *Leishmania* [28, 68], though knowledge on the development of *Mundina* in invertebrates is limited.

**Fig. 7:**
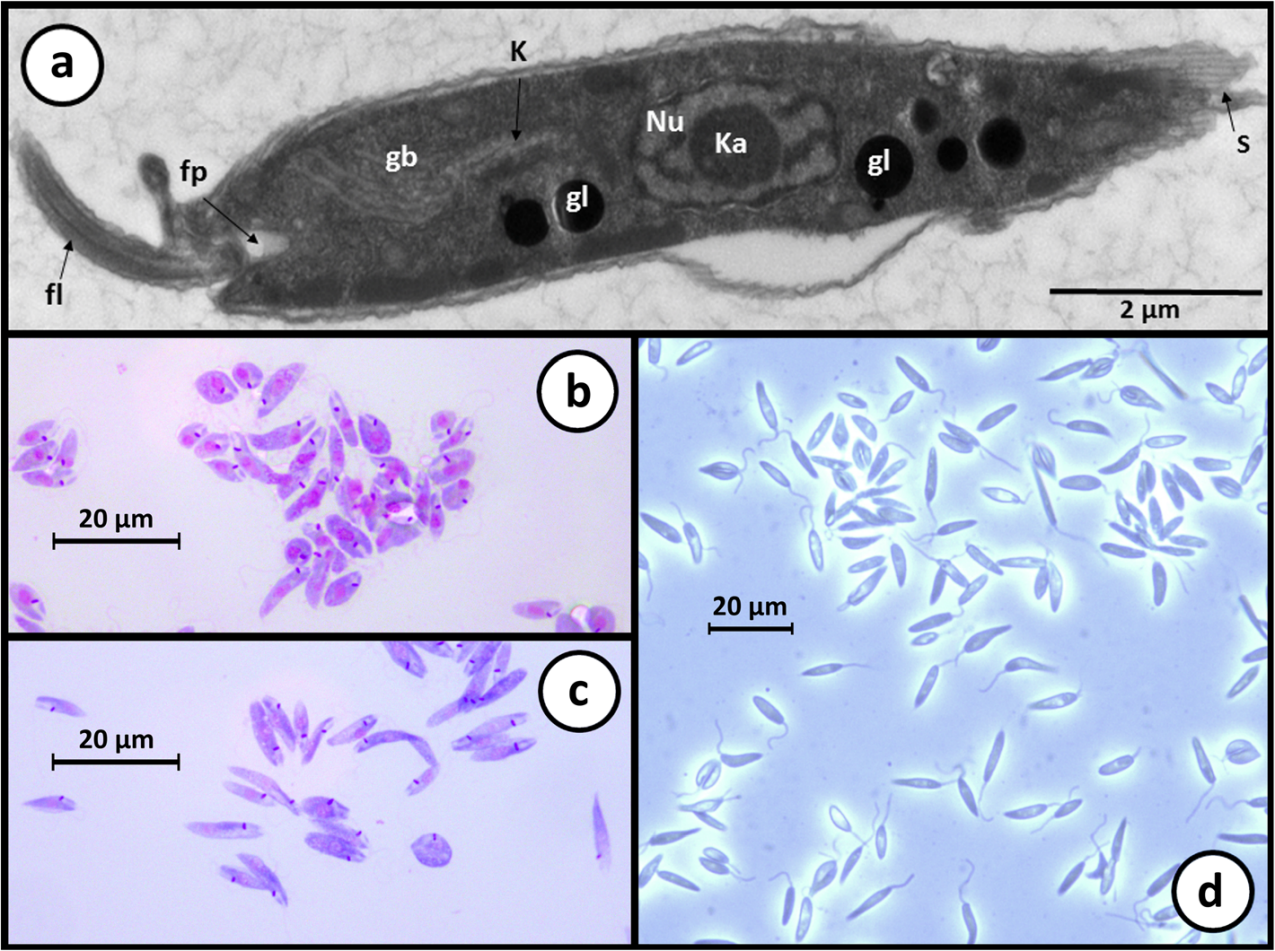
Light and electron micrographs of *Zelonia australiensis.* **a** Transmission electron micrograph showing the gross morphological features of *Zelonia australiensis* promastigotes including the nucleus (Nu), karysome (Ka), kinetoplast (K), flagella (fl), flagella pocket (fp), glycosomes (gl) and the Golgi body (gb). Subpelicular microtubules (S) give some cell edges a striated appearance, depending on the angle of sectioning. **b**, **c** Light micrographs showing promastigotes in a *Leishman* stained smear. **d** Light micrograph of a live-cell wet preparation viewed under phase contrast microscopy

In Peripylaria development begins following attachment of parasites to the wall of the sand fly pylorus and ileum (i.e. the hindgut), followed by movement into the midgut and final invasion of the foregut [65, 66]. This section comprises important pathogens of humans and other mammals, including all *Viannia* parasites and two saurian species; *L.* (*S*.) *adleri* and *L.* (*S.*) *tarentolae* [65, 69]. Members of the Suprapylaria section are limited to growth and differentiation in the midgut and foregut of the sand fly [69], and comprise of the phylogenetically supported subgenus *Leishmania* and the vast majority of medically significant species [66].

The protein phosphoglycan-rich promastigote secretory gel (PSG) plays a crucial role in *Leishmania* development within the sand fly by conditioning the gut for differentiation from poorly infective procyclics into highly infective metacyclic promastigotes, a process referred to as metacyclogenesis [70–72]. This PSG is secreted by leptomonad promastigotes (Fig. 5) and creates a blockage in the sand fly gut within a week after infection, which is an essential component of *Leishmania* transmission. The PSG plug forms in the anterior midgut, stomodeal valve and foregut and makes feeding difficult, causing the fly to take prolonged and multiple blood meals from the same host. The plug likely generates backpressure in the gut that causes dislodgement and regurgitation of the plug into the bite wound, along with highly infective metacyclic promastigotes [23, 73]. The formation of a PSG plug is best known in phlebotomine sand flies, though a mass resembling a PSG plug was also observed in *Forcipomyia* (*Lasiohelea*) species midges infected with *Leishmania macropodum* [23, 68].

## Problems with classical trypanosomatid systematics

### Dixenous and monoxenous clades are paraphyletic

The classical trypanosomatid taxonomic system based on morphology and life-cycles was established in the 1960s by Hoare and Wallace, who redefined kinetoplastid systematics [26, 74, 75]. Early descriptions defined *Leptomonas* as having a life-cycle containing both promastigote and amastigote stages [76], being parasitic only to invertebrates and of no medical importance [77–79]. This system also restricted *Leptomonas* spp. and other monoxenous genera to a single invertebrate host. This paradigm is no longer accepted as molecular studies have confirmed that many trypanosomatids parasitise multiple insect species [54]. *Leptomonas* along with other monoxenous genera (Table 1) are often described as “lower trypanosomatids” under the assumption that all trypanosomatids are thought to share a single monoxenous ancestor [80]. While the phrase “lower trypanosomatid” remains in general use, the concept is imperfect as dixenous parasitism has evolved independently multiple times in trypanosomatids, meaning that some dixenous genera (e.g. *Trypanosoma*) are basal to certain monoxenous species (e.g. *Crithidia*) [27, 81]. Regardless, it is generally accepted that monoxenous parasitism represents the more primitive, ancestral state as monoxenous species are typically thought to be more numerous and diverse [24, 82]. This does assume however that all trypanosomatid species evolve at the same rate which is thought not to be the case [26, 67]. Regardless, the presumably monoxenous trypanosomatid *Paratrypanosoma confusum* does support this concept as it invariably occupies the most basal position in molecular phylogenies of trypanosomatids [83].

While monoxenous trypanosomatids are considered non-pathogenic to vertebrates, their restriction to invertebrates is not absolute considering the reports of monoxenous trypanosomatids (e.g. *Leptomonas seymouri*) exploring the dixenous niche [80, 84–86] (discussed below). While these cases are seemingly rare exceptions rather than common events, they highlight that isolation of promastigotes from a vertebrate does not confirm a *Leishmania* infection which does confound classic taxonomic definitions.

## Exploration of the dixenous niche by monoxenous parasites

Of the 19 trypanosomatid genera currently recognised, fourteen are considered monoxenous, parasitising one or more insect hosts [27, 28, 87] (Table 1). Whilst they are considered non-pathogenic to vertebrates, reports of infections with monoxenous species resulting in human illness date back to the 1980s [34, 88] (Table 2). A common trend in many cases was the presence of an immunocompromised state, generally resulting from HIV co-infection [84, 89]. Patients co-infected with HIV and a monoxenous trypanosomatid may present with skin lesions, splenomegaly and other symptoms resembling VL and/or CL [37, 80, 90]. Several cases of infection with *Leptomonas* spp. have recently come to prominence in HIV negative Indian Kala-azar (VL) patients infected with *Leishmania donovani* [53, 80, 85, 91]. Whilst the impact of *Leptomonas* co-infection on the clinical outcome of Kala-azar in these patients remains unexplored, these events confound classic taxonomic definitions and have even led to the incorrect assignment of *Leptomonas* DNA sequences to *L. donovani* [85]. The first co-infection involving *Leishmania donovani* and a monoxenous trypanosomatid was reported in 2010 by Srivastava et al. [91], who employed DNA sequencing to confirm *L. donovani* infection concurrent with a trypanosomatid designated as *Leptomonas* sp. BHU [91]. Several similar cases have been reported since, with later studies incriminating *Leptomonas seymouri* as the opportunistic monoxenous agent [80, 85, 90]. In such cases, *Leptomonas* may be detected in bone marrow and splenic aspirates, reminiscent of VL [90]. It was theorised that VL induces strong immunosuppression providing the opportunity for *L. seymouri* to infect these patients [37, 84]. *Leptomonas* was once considered a primitive sister taxon to *Leishmania*. In actual fact these genera exhibit relatively little divergence, so it is not unprecedented that *L. seymouri* might occasionally explore the dixenous niche and possess adaptations that allow it to do so [53, 80]. Another point of contention is that *L. seymouri* was originally isolated from a strictly phytophagous *Dysdercus suturellus* “cotton stainer” bug (family Pyrrhocoridae) [53], raising questions surrounding the route of infection in cases of *L. donovani* - *L. seymouri* co-infections. Rinsing wounds or sores with water contaminated with *L. seymouri* is a possibility, and rinsing of syringes in water contaminated with trypanosomatids by intravenous drug abusers is another alternative [84], though drug abuse was not noted in the Indian VL cases. A global study screening the Pyrrhocoridae for trypanosomatids failed to identify *L. seymouri* parasitising this group [53, 92], suggesting that *L. seymouri* may possess an alternative hematophagous host.

**Table 2:** Historical overview of the studies describing unusual infections caused by monoxenous trypanosomatids

Year	Reference	Description
1980	McGhee & Cosgrove [88]	Report of a possible monoxenous infection in a woman from Texas presenting with ill-defined symptoms. Examination of the cultures excluded the possibility of *Leishmania* or *Trypanosoma* infection, and suggested the organism was a *Herpetomonas* sp.
1986	Githure et al. [143]	Trypanosomatids isolated from HIV-negative patients in Kenya were revealed to be more closely related to *Crithidia* species through iso-enzyme and kDNA analyses, than that of *Leishmania.*
1988	Morsey et al. [93]	An unnamed trypanosomatid species was isolated from rats and stray dogs in Egypt in 1989 by Morsey et al. (1988). The rodent/canine isolate was later found to be a member of the genus *Herpetomonas* [94].
1989	Conchon et al. [144]	*Crithidia ancanthocephali*, *Crithidia fasciculate*, *Crithidia thermophile*, *Leptomonas seymori*, *Herpetomonas samuelpessoai* were used to experimentally infect tomato plants (*Lycopersicon esculentum*).
1991	Sabbatani et al. [145]	A case of unusual visceral leishmaniasis was reported in a HIV-positive 10-year-old girl from Guinea-Bissau, where the disease had not been previously identified. It was speculated that this case resulted from an infection with a reptilian trypanosomatid that had not been identified in humans previously.
1992	Mebrahtu et al. [146]	Parasites isolated from HIV-negative patients suffering from visceral leishmaniasis were described as resembling *Crithidia* species rather than the pathogenic *Leishmania*.
1996	Jimenez et al. [147]	An “unusual *Leishmania*-like parasite” was reported in a case of visceral leishmaniasis/HIV co-infection.
1998	Pacheco et al. [89]	A monoxenous trypanosomatid was isolated from the bone marrow of an HIV patient presenting with a visceral leishmaniasis-like syndrome. The patient was positive for a *Leishmania braziliensis* infection. Molecular analyses also revealed a co-infecting parasite that did not belong to the genus *Leishmania* or *Trypanosoma* and hybridisation analyses confirmed kinetoplast DNA (kDNA) cross-hybridisation with *Leptomonas pulexsimulantis*.
2007	Srivastava et al. [91]	Report of nine cases of visceral leishmaniasis in patients from India. PCR analysis revealed the presence of both *Leishmania donovani* and a *Leptomonas* species designated *Leptomonas* sp. BHU. It was proposed that the monoxenous flagellates were able to infect these patients due to immune-suppression associated with visceral leishmaniasis.
2012	Ghosh et al. [85]	In a study of Indian *Leishmania donovani* infected patients, 4/29 (13.8%) patients with visceral leishmaniasis and post-kala-azar dermal leishmaniasis (PKDL) were co-infected with the previously identified *Leptomonas* sp. BHU, which was later confirmed as *Leptomonas seymouri*.
2013	Singh et al. [80]	Through whole genome sequencing of the *L. donovani* clinical isolates from India, the presence of monoxenous trypanosomatids in cases of visceral leishmaniasis was reported as *Leptomonas seymouri*. It is important to note that a recent study by Kraeva et al. found that some of the DNA sequences of *Leptomonas seymouri* were misidentified as *Leishmania donovani* in GenBank [53].

*Note*: A case of diffuse cutaneous infection caused by a presumably monoxenous parasite was reported in 1995 in an immunocompromised patient infected with HIV [148]. The same parasite isolated in 1995 by Dedet et al. was reported in an immunocompromised patient from Martinique causing a localised cutaneous lesion [149]. This parasite has since been confirmed as *Leishmania martiniquensis* [150] and so has been excluded from the table

Infections with ‘monoxenous’ trypanosomatids have also been reported in vertebrates other than humans [86]. For example, an unnamed trypanosomatid was isolated from rats and stray dogs in Egypt in 1989 by Morsey et al. (1988) [93]. A later study by Podlipaev et al. [94] confirmed this parasite as a member of the ‘monoxenous’ genus *Herpetomonas*, and a very close relative of *Herpetomonas ztiplika*; a parasite that infects biting midges [94, 95].

## Trypanosomatid morphology

Light and electron microscopic studies show that trypanosomatids possess a general ultrastructure that is universally conserved across the family [32]. Trypanosomatids possess an interlaced network of circular DNA known as the kinetoplast DNA (kDNA), associated with the base of a single flagellum that is attached to a slender cell body [32]. Recently, Wheeler et al. [32] conveniently divided trypanosomatids into two distinct morphological superclasses that are supported by molecular phylogeny; the ‘juxtaform’ superclass which includes epimastigote and trypomastigote morphotypes, defined by the presence of a laterally attached flagellum (e.g. *Trypanosoma*), and the ‘liberform’ superclass which possess a free flagellum (e.g. *Leishmania* and *Leptomonas*), which includes the opisthomastigote, choanomastigote and promastigote morphotypes [32] (Fig. 2). The spherical amastigote morphotype of trypanosomatids occurs in both morphological superclasses [32]. Therefore, the presence or absence of amastigotes cannot be used to inform evolutionary relationships. Along with the morphotypes described above, several others have been defined [26].

Liberform trypanosomatids such as *Leptomonas*, *Zelonia* (Fig. 7) and *Leishmania* predominately exist as promastigotes in standard culture, though axenic amastigotes of *Leishmania* can be induced in vitro [96]. Regardless, these species are highly pleomorphic, shifting between morphotypes depending on their growth phase, host, and host compartment they are occupying [32, 34, 68, 87, 97, 98]. Promastigotes of *Leishmania* are divided into five morphological categories which include: (i) procyclic promastigotes - the replicating form in the sand fly; (ii) nectomonad promastigotes - the elongated promastigote stage; (iii) haptomonad promastigotes - a stage possessing a disc-like expansion at the flagellar tip; (iv) leptomonad promastigotes - the stage that secretes PSG and is a precursor to; (v) metacyclic promastigotes - the form infective to the vertebrate host (Fig. 5) [70].

Generally, the trypanosomatid morphotypes are distinguished by their cell shape, the relative position of their nucleus to the kinetoplast [33], and flagellum positioning and attachment to the cell body [32, 74, 75]. These and other subtleties once served as taxon-defining characteristics under the classic system, and are now considered to a very minor extent given the inadequacies of this system revealed by genetics [26, 68, 97]. Phylogenetic analyses have culminated in the renaming of several species due to various instances of polyphyly introduced by the classic system, and are now considered mandatory for making accurate taxonomic assignments for new isolates [27, 28, 97, 99, 100].

As new trypanosomatid species are discovered, discernible features that were once taxon defining are becoming less suitable for this purpose. The discovery and description of *Novymonous esmeraldas* and characterisation of its bacterial endosymbiont provides a recent example [87]. Bacterial endosymbionts were once confined to the Strigomonadinae which occupy a single phylogenetic clade [26]. However, *N. esmeraldas* is a distantly related trypanosomatid of the subfamily Leishmaniinae, making bacterial endosymbiosis a polyphyletic trait. However, the endosymbionts of the Strigomonadinae are only distantly related to those of *Novymonas*, suggesting these relationships developed independently in the two trypanosomatid subfamilies [87]. In any case, the main confusion with trypanosomatid systematics lies in the discordance between their visually discernible characteristics and their molecular biology.

## Different genera, different rules

Advances in molecular biology and phylogenetics have revealed a lack of taxonomic concordance between gene sequence similarity and species demarcation, most notably within the clinically relevant genera *Leishmania* and *Trypanosoma* [7]. For example, small subunit ribosomal RNA (SSU rRNA) sequences of *T. cruzi* and *T. brucei* are separated by a genetic distance of approximately 12% while different species within the genus *Leishmania* possess SSU rRNA sequences separated by a distance of less than 1% in some cases [101]. Similarly, *T. cruzi* isolates can be separated into several discrete typing units [102, 103], which could easily constitute different species if they were held to the same species demarcation criteria as the genus *Leishmania*. This problem has been raised by previous investigators, who offer sensible solutions to this issue, including delineation of new species based on a 90% sequence similarity threshold [27].

## Hybridization between species

Based on current understanding *Leishmania* possesses a sexual or parasexual cycle that allows recombination between distinct lineages, and in some cases different species [80]. Instances of hybridisation have been known from studies of trypanosomatid field isolates for nearly two decades [104]. A study involving isoenzyme analysis and molecular karyotyping of two *Leishmania* strains isolated from wild animals in Saudi Arabia identified a suspected hybrid isolate distinct from other *Leishmania* species, possessing characteristics of both *Leishmania major* and *Leishmania arabica* [105]. Banuls et al. [106] identified suspected *Leishmania* hybrids in stocks isolated from humans in Ecuador, representing potential crosses between *L. braziliensis* and *L. panamensis*/*guyanensis*. More recently, whole genome sequencing was used to confirm hybridisation in 11 unique isolates of *Leishmania infantum* from sand flies and one CL patient [107]. Additionally, crosses between *L. major* and *L. infantum* have been achieved under experimental conditions [108]. The limited genetic divergence between *Leishmania* species (discussed above), in conjunction with reports of hybridization, indicate that species delineation in this genus could be relaxed. However, echoing the points made by previous investigators [27], collapsing the many *Leishmania* species into a few would only generate confusion, particularly for clinicians. The grouping of *Leishmania* species into phylogenetically supported subfamilies as per Espinosa et al. [28] seems an elegant solution as hybridization seems to only occur within subfamilies and not between them, though this requires further investigation.

## Exploration of alternative vectors

Cases of locally acquired Australian cutaneous leishmaniasis were first reported in 2004, in captive red kangaroos (*Macropus rufus*) from a wildlife park near Darwin, in Australia’s Northern Territory [20, 109]. In 2009, the same species was isolated from three additional macropod species, *Macropus robustus woodwardi* (northern wallaroo), *Macropus bernardus* (black wallaroo) and *Macropus agilis* (agile wallaby) [22]. This was the first report confirming Australia’s endemicity for leishmaniasis, albeit a form restricted to native animals, with no evidence for human infection. Of the 18 known Australian phlebotomine species, little is known about their biological capacity as vectors of leishmaniasis and *Leishmania* has never been detected in these species [23, 110]. Australian phlebotamines are thought to feed solely on small mammals, birds and reptiles which led to the speculation that this species (recently dubbed *Leishmania macropodum* [68]), might be transmitted *via* an alternative vector [22]. Later investigations identified a day feeding midge, *Forcipomyia* (*Lasiohelea*) (Diptera: Ceratopogonidae), as the likely vector [23]. *Leishmania macropodum* was prevalent in ~15% of *Forcipomyia* (*L.*) midges tested and similar patterns of promastigote migration to the midgut were observed between *Forcipomyia* and *Leishmania*, when compared to those observed in the *Leishmania*-phlebotomine interaction [23]. In addition to *Forcipomyia*, other insects may be capable of supporting *Leishmania* replication (at least temporarily) based on evidence from experimental infections and molecular evidence from naturally infected insects. The biting midge *Culicoides sonorensis* (Diptera: Ceratopogonidae) is capable of developing late stage infections with *Leishmania enriettii* [111]. *Culicoides nubeculosus* supported *L. infantum* infection for up to 7 days post blood meal [72]. *Leishmania infantum* DNA has also been detected in naturally infected *Culicoides* spp. collected in Tunisia [112], while DNA from *Leishmania amazonensis* and *Leishmania braziliensis* was detected in naturally infected *Culicoides* spp. from Brazil [113]. These studies call into question the exclusivity of the *Leishmania*-phlebotomine interaction, particularly in the case of the Australian species which naturally infects *Forcipomyia* and reportedly produces a PSG plug in this insect [23].

## The phylogenetic solution and its caveats

Phylogenetic evidence has confirmed on multiple occasions that naming trypanosomatid taxa based on classic systems often gives rise to polyphyly [99, 114]. This is due to divergence in DNA sequences that give rise to few changes in morphology, host range and other biological characteristics [7, 97]. The absence of adequate boundaries relating to morphological and host-based criteria highlight the requirement for a phylogenetic solution to trypanosomatid taxonomy [115]. Estimating rates of evolutionary divergence is attributed to Zuckerkandl and Pauling’s concept of the molecular clock which suggests that differences between amino acid sequences are relatively proportional to evolutionary events of divergence [116–118]. This approach assumes that rates of genetic change are constant amongst species of common descent, allowing estimates of rates to be extrapolated across phylogenetic trees [119]. Subsequent studies suggested that if these clocks could infer evolutionary timescales, the differences between sequences must be exclusive to sites of neutrality to equal the rate of mutation, supporting Kimura’s theory of neutral molecular evolution [120, 121].

Slow evolving (SE) genes are characterised by a slower divergence rate, undergoing fewer nucleotide substitutions over time. Sequences of SE genes are most suitable for investigating evolutionary relationships over larger time-scales [122]. Phylogenetic studies using only the small subunit ribosomal RNA (SSU rRNA) genes may not provide reliable phylogenetic inference for species within the same taxonomic family for example, as the rRNA genes evolve very slowly [123, 124]. Instead, a middle ground must be reached for resolving relationships between closely related organisms. Several housekeeping genes encoding proteins involved in basic cellular functions have provided the most useful information on relationships between the various *Leishmania* species [125]. Gene sequences of the RNA polymerase II largest subunit (RPOIILS) [97, 123], DNA polymerase α catalytic polypeptide (POLA) [123], glyceraldehyde-3-phosphate dehydrogenase (GADPH) [18, 97], heat-shock protein 20 [125], and heat-shock protein 70 [126], are preferred targets for analysing phylogenetic relationships between *Leishmania* species and their close relatives.

Despite the power of phylogenetic inference there are some important caveats to be considered. For example, the structure of phylogenetic trees can change markedly depending on the locus used, affecting the way relationships between taxa are interpreted. Using concatenated sequences from multiple phylogenetically informative loci has been suggested as a means to counteract this problem and improve the robustness of phylogenetic trees [27]. Similarly, the structure of phylogenetic trees can change markedly depending on the outgroup selected, or if certain taxa are included or excluded from the analysis [27]. The robustness of phylogenetic predictions is also dependent on the number of appropriate taxa included in the analysis. An important example involves the early debate surrounding the most basal *Leishmania* subgenus (touched upon previously herein) [19, 67, 68, 127–130]. This was considered pertinent to selection of the most appropriate outgroup, and would have implications relating to *Leishmania* biogeography and even its first vertebrate hosts. If the *Sauroleishmania* were the earliest branching group, this would implicate reptiles as the original vertebrate host of an ancestral *Leishmania* parasite. If *Viannia* were most basal, *Leishmania* probably evolved in the Neotropics [128]. With the addition of several other taxa, including some novel monoxenous genera that represent appropriate outgroups; phylogenetic evidence indicates that *Mundinia* is the most basal *Leishmania* subgenus [28, 68].

Discrepancies also arise when using single (or very few) trypanosomatid isolates to define new genera and establish taxonomic assignments. *Zelonia costaricensis* represents one such example. Originally assigned to the genus *Leptomonas* [131], the inclusion of several novel trypanosomatid isolates in phylogenies confirmed that *Zelonia* is distinct from the *Leptomonas*, *Lotmaria* and *Crithidia* clade, but is immediately basal to the *Leishmania*, *Endotrypanum* and *Porcisia* clade, warranting establishment of a new genus [28, 68]. A solution exemplified by Espinosa et al. [28] is to obtain sequence data from multiple sister isolates of a new candidate taxon before making new assignments. This will ensure that trypanosomatid taxa remain monophyletic by providing robust phylogenetic support.

Two recent studies used phylogenetics to approximately date the origin of the first ancestral *Leishmania* parasites with intriguing results [68, 132]. However, this type of analysis is complicated by the fact that closely related taxa may evolve at different rates due to environmental pressures, including those exerted by the host immune response. These pressures are of course markedly different for monoxenous and dixenous taxa. The accuracy of these analyses also relies on selection of a precise calibration point; that is, an accurately dated geological event (or other) that is known to have triggered a speciation event in closely related taxa. As the accuracy of these predictions are difficult to test, vicariance events dated using phylogenetic inference should be considered approximations at best.

## Conclusions

Important developments have been made in the field of trypanosomatid taxonomy in recent years, facilitated by the identification of novel trypanosomatid species in addition to advances in molecular biology and phylogenetics. Phylogenetic inference has identified flaws in the classical system of trypanosomatid taxonomy which relied predominantly on parasite morphology and host preferences. Despite significant sequence divergence, trypanosomatids have experienced limited morphological change throughout their evolution such that the subtle morphological variations that exist do not reflect molecular phylogeny. Phylogenetics has corrected some of these issues by supporting the assignment of some trypanosomatids to different genera and establishment of new genera, ensuring that taxa remain monophyletic [27]. Despite these advances, trypanosomatid systematics still remains imperfect. There remains a lack of consensus for delineating species based on molecular divergence, and the limits differ markedly for *Trypanosoma* and *Leishmania*. While not recommended due to the confusion it would cause, molecular evidence probably supports the collapsing of some *Leishmania* species into one, or splitting *Trypanosoma* into different species or even different genera depending on the limits of molecular divergence applied. Additionally, some trypanosomatid genera (i.e. *Leptomonas*) remain paraphyletic [68, 133]. Transmission of *L. macropodum* by a day feeding midge rather than a phlebotomine sand fly and the detection of clinically important *Leishmania* species in hematophagous insects such as *Culicoides* spp., questions the exclusivity of the *Leishmania*-sand fly relationship. Reports of monoxenous trypanosomatid infections in vertebrates and the grouping of presumably monoxenous trypanosomatids (e.g. *Zelonia* and *Novymonas*) in dixenous clades occupied by *Leishmania* and *Endotrypanum*, also call into question the suitability of host relationships as a taxonomic criterion. Long accepted dogmas such as the ‘one-insect-one-parasite’ rule have been overturned, supported by molecular evidence. These examples highlight the importance of employing molecular biology and phylogenetics when making taxonomic assignments relating to trypanosomatids. The continued isolation and characterisation of novel trypanosomatids will ensure this field continues to develop, by allowing resolution of evolutionary relationships between members of this group with increasing accuracy. To this end, generation of high-quality genome sequences for additional species would be of huge benefit, allowing relationships to be phylogenetically scrutinised across numerous loci. The genomes of trypanosomatids that encroach on the monoxenous-dixenous boundary (e.g. *Zelonia* and *Novymonas*) may prove instrumental to understanding the path to dixenous parasitism by providing new knowledge on the innovations that evolved to allow this transition [58].

## Abbreviations

CL: Cutaneous leishmaniasis
GAPDH: Glyceraldehyde-3-phosphate dehydrogenase
Hsp20: Heat-shock protein 20
Hsp70: Heat-shock protein 70
MCL: Mucocutaneous leishmaniasis
POLA: DNA polymerase α catalytic polypeptide
PSG: Promastigote secretory gel
RPOIILS: RNA polymerase II largest subunit
SE: Slow evolving
SSU rRNA: Small subunit ribosomal RNA
VL: Visceral leishmaniasis
